# Decoding Sepsis: A 16-Year Retrospective Analysis of Activation Patterns, Mortality Predictors, and Outcomes from a Hospital-Wide Sepsis Protocol

**DOI:** 10.3390/jcm14165759

**Published:** 2025-08-14

**Authors:** Marcio Borges-Sa, Andres Giglio, Maria Aranda, Antonia Socias, Alberto del Castillo, Joana Mena, Sara Franco, Maria Ortega, Yasmina Nieto, Victor Estrada, Roberto de la Rica, Son Llatzer’s Multidisciplinary Sepsis Unit

**Affiliations:** 1 Multidisicplinary Sepsis Group, Son Llatzer Hospital, 07198 Palma, Spain; agiglioj@gmail.com (A.G.); marandacorreo@gmail.com (M.A.); sociasmir@gmail.com (A.S.); albertodcb@hotmail.com (A.d.C.); jmena@hsll.es (J.M.); sfranco@hsll.es (S.F.); mery_6666@hotmail.com (M.O.); yasmina.nieto@hsll.es (Y.N.); 2 Intensive Care Unit, Son Llatzer Hospital, 07198 Palma, Spain; 3 Health Research Institute of the Balearic Islands (IdISBa), 07120 Palma, Spain; roberto.delarica@gmail.com; 4 Faculty of Medicine, Balearic Islands University, 07122 Palma, Spain; 5 Fundación Codigo Sepsis, 46003 Valencia, Spain; 6 Critical Care Center, Clínica Las Condes Hospital, Santiago 7591046, Chile; 7 Critical Care Department, Faculty of Medicine, Finis Terrae University, Santiago 7640471, Chile; 8 Informatics Department, Son Llatzer Hospital, 07198 Palma, Spain; vestrada@hsll.es

**Keywords:** sepsis, septic shock, protocol activation, early detection, mortality predictors, resource utilization, hospital wide

## Abstract

**Background:** Sepsis remains a leading cause of mortality in hospitalized patients. We evaluated characteristics and outcomes of patients identified through a comprehensive hospital-wide sepsis protocol over a 16-year period. **Methods:** This retrospective cohort study analyzed hospital-wide sepsis protocol activations at a tertiary care hospital in Spain from 2006 to 2022. The protocol required at least two SIRS criteria plus evidence of organ dysfunction in patients over 14 years old. We analyzed demographics, activation criteria, hospital location, mortality predictors using univariate and multivariate analyses, including propensity score modeling, and resource utilization trends. **Results:** A total of 10,919 patients with 14,546 protocol activations were identified. The median age was 69 years (IQR: 56–78), with 60.9% male patients. Protocol activations occurred in the emergency department (54%), ICU (34.2%), and inpatient wards (11.8%). The most common SIRS criteria were tachycardia (75.6%), tachypnea (50.4%), and fever (48.5%). Prevalent organ dysfunctions included hypotension (53%), hypoxemia (50.1%), oliguria (28.9%), and altered mental status (22%). Overall in-hospital mortality showed a significant linear downward trend from 26.5% in the first year to 13.6% in later years (*p* < 0.01). Propensity score analysis confirmed independent mortality predictors included hyperlactatemia (aOR 2.21), altered consciousness (aOR 2.09), hypotension (aOR 1.87), and leukopenia (aOR 1.79). ICU admission rate decreased from 58% to 24% over the study period. **Conclusions:** This 16-year analysis shows that comprehensive hospital-wide sepsis protocols achieve sustained mortality reduction with improved resource utilization efficiency. These findings support implementing comprehensive sepsis protocols as an effective strategy for improving sepsis outcomes.

## 1. Introduction

Sepsis remains a critical global health challenge, affecting approximately 49 million individuals annually and causing an estimated 11 million deaths worldwide [1]. In hospital settings, sepsis accounts for 30–50% of all deaths, with mortality rates ranging from 15% to 30% for sepsis and 40% to 60% for septic shock [2]. Despite significant advances in critical care medicine, the incidence of sepsis continues to rise, driven by aging populations, increasing antimicrobial resistance, more immunosuppressed patients, the rising complexity in surgical interventions, and improved recognition [3]. The economic burden is equally substantial, with sepsis care consuming more than $62 billion annually in the United States alone, representing the most expensive condition treated in hospitals [4].

Early recognition and prompt intervention are crucial to improve sepsis outcomes, as each hour of delay in appropriate antibiotic therapy is associated with an approximate 7.6% increase in mortality [5]. However, sepsis management faces multiple difficulties, including different clinical and laboratory phenotypes, delays in recognition, inconsistent application of evidence-based practices, and heterogeneity in organizational approaches across different healthcare settings [6,7]. These challenges highlight the need for standardized, systematic approaches to sepsis care that can overcome barriers to timely intervention and reduce the substantial mortality and resource utilization associated with this syndrome [8].

Recent advances in sepsis biomarkers, particularly procalcitonin (PCT), monocyte distribution width (MDW), and presepsin (PSP), have shown promise in improving sepsis recognition and patient outcomes [9,10,11]. These emerging tools complement clinical assessment and may enhance the effectiveness of structured sepsis protocols, though their integration into routine clinical practice varies across healthcare settings.

In response to these obstacles, structured sepsis codes, protocols, and specialized units have emerged as organizational strategies to improve sepsis outcomes [8]. These approaches aim to standardize sepsis identification and management through systematic screening, rapid response teams, protocol-driven interventions, and comprehensive care pathways [2,12]. Previous studies have demonstrated that implementation of sepsis protocols can reduce mortality by 15–30% and decrease resource utilization through earlier intervention and standardized care [8,13]. Additionally, the development of dedicated sepsis teams—comprising critical care physicians, specialized nurses, pharmacists, and infectious disease specialists—ensures that sepsis management is timely, consistent, and adherent to evidence-based practices [14]. However, long-term evaluations of comprehensive sepsis programs spanning multiple years are scarce, limiting our understanding of their sustained impact on patient outcomes and hospital-wide performance metrics.

This study aimed to analyze a large retrospective cohort of sepsis protocol activations at a tertiary care hospital over a 16-year period (2006–2022), evaluating the evolution and impact of a structured sepsis program on patient outcomes and resource utilization. By examining sepsis protocol activations, we sought to characterize the demographic and clinical features of this population, identify patterns of protocol activation across hospital settings, analyze the evolution of key outcome measures over time, and determine factors associated with mortality and resource utilization. This comprehensive assessment of a sustained sepsis program provides valuable insights into the long-term impact of systematic sepsis management strategies in a real-world clinical setting.

## 2. Materials and Methods

### 2.1. Study Design and Setting

This retrospective cohort study was conducted at Son Llatzer University Hospital, a 440-bed tertiary care center in Spain, analyzing data collected from January 2006 to December 2022. The hospital is a high-complexity center serving a population of approximately 300,000 people and includes most medical and surgical specialties, though notably excluding neurosurgery and cardiac surgery. The study was designed to evaluate the impact of a structured sepsis protocol on patient outcomes over a 16-year period.

### 2.2. Sepsis Protocol Activation System

A comprehensive, computerized sepsis protocol alert system was developed and implemented across all hospital areas (excluding pediatrics). The protocol was designed for the early identification of severe sepsis and septic shock according to Sepsis-2 criteria. Key components of the protocol activation system included:

1. Standardized screening and activation criteria based on systemic inflammatory response syndrome (SIRS) parameters and evidence of organ dysfunction;
2. An electronic protocol that could be activated by any physician throughout the hospital upon recognition of suspicious cases;
3. A centralized alert system with automated notification of potential sepsis cases during the first 72 h;
4. Laboratory and microbiological alert integration;
5. An electronic documentation system for protocol activations.

The protocol activation system was accessible online to all hospital physicians, allowing for immediate notification upon recognition of patients meeting sepsis criteria. Throughout the study period, ongoing periodical educational activities were conducted to maintain awareness of sepsis recognition criteria and the importance of early activation, including general hospital sessions, department-specific training, infographics, and separate education programs for physicians and nurses [15].

### 2.3. Patient Selection and Data Collection

The study included adult patients (≥14 years) who met criteria for activation of the sepsis protocol in all hospital areas: emergency department (ED), inpatient wards, and intensive care unit (ICU). Sepsis protocol activation required the presence of at least two SIRS criteria plus evidence of at least one organ dysfunction, equivalent to severe sepsis or septic shock according to the Sepsis-2 definitions. The code could be activated voluntarily by clinicians in charge of the patient or by the multidisciplinary sepsis unit (MSU).

All cases were evaluated prospectively by the MSU, a multidisciplinary team of physicians and nurses, in charge of both critical care and internal medicine specialists dedicated to infectious diseases and sepsis care and including physicians and clinical personnel from all relevant areas, such as ED or surgical specialties.

Patients were identified through the hospital’s automatic detection systems based on electronic health records, treating physician consultations, and a telephone alert system from the microbiology department when positive blood cultures or other microbiological results were detected.

Data collected at the time of protocol activation included:

1. Demographic information: Age and sex;
2. Clinical parameters at activation: SIRS criteria met (temperature, heart rate, respiratory rate, white blood cell count);
3. Organ dysfunction criteria at activation: Hypotension, hypoxemia, altered mental status, oliguria, elevated creatinine, coagulopathy, hyperbilirubinemia, and hyperlactatemia;
4. Sepsis classification: Severe sepsis (sepsis to our protocol) vs. septic shock according to SEPSIS-2 definition;
5. Activation details: Time of day, hospital location at activation, and department initiating activation;
6. Resource utilization outcomes: ICU admission, length of stay in hospital, and ICU;
7. Clinical outcomes: In-hospital mortality and ICU mortality.

### 2.4. Sepsis Definitions

Sepsis-2 definitions were used consistently throughout the entire study period (2006–2022) to maintain temporal comparability and avoid selection bias. The protocol was specifically designed for early sepsis identification using SIRS criteria plus organ dysfunction, which enables earlier patient detection compared to SOFA-based approaches that may delay interventions until a SOFA score ≥2 is achieved.

### 2.5. Definitions

Throughout the study period, consistent definitions were maintained [16] (Table 1):

### 2.6. Statistical Analysis

Descriptive statistics were used to summarize patient characteristics and outcomes. Continuous variables were presented as median with interquartile range (IQR) or mean with standard deviation (SD), depending on data distribution. Categorical variables were presented as frequencies and percentages.

Temporal trends in protocol activations, activation criteria, presenting features, and outcomes over the 16-year period were assessed in the overall results, as well as specific analysis considering services hospital-wide. Comparisons between years were performed using appropriate statistical tests. For categorical variables, chi-square or Fisher’s exact test was used, while continuous variables were analyzed using the Mann–Whitney U test or Kruskal–Wallis test.

Factors associated with mortality were identified using univariate analysis. Variables with *p* < 0.10 in univariate analysis were included in multivariate logistic regression models to determine independent predictors of mortality, adjusting for age, sex, and sepsis severity. Results were expressed as adjusted odds ratios (aOR) with 95% confidence intervals.

All statistical analyses were performed using XLSTAT 2024.1 statistical software, with statistical significance defined as *p* < 0.05.

### 2.7. Missing Data Management

Missing data were minimal across the study variables. Variables with >20% missing data were excluded from multivariate analysis to ensure model reliability. For included variables, complete case analysis was performed given the low percentage of missing observations.

### 2.8. Ethical Approval

This study forms part of the PIMIS database analysis (Computerized Multidisciplinary and Integral Sepsis Protocol), approved for review and publication by the ethics committee of the Balearic Islands (CEIC ID: IB 5320/23 PI). As a retrospective analysis of anonymized data collected as part of routine clinical care, individual patient consent was waived.

### 2.9. Declaration of Generative AI and AI-Assisted Technologies in the Writing Process:

During the preparation of this work, the authors used Paperpal to improve the readability of this paper. After using this tool, the authors reviewed and edited the content as needed and take full responsibility for the content of the publication.

## 3. Results

### 3.1. Patient Characteristics and Protocol Activation Patterns

A total of 10,919 individual patients with 14,546 sepsis protocol activations were included in the study over the 16-year period. The median age of the entire cohort was 69 years (IQR: 56–78), and 60.9% of patients were male.

### 3.2. SIRS, Organ Dysfunction Criteria and Severity at Activation

The analysis of SIRS criteria present at the time of protocol activation revealed that the median SIRS criteria at activation was 3 (IQR 2–4). Tachycardia was the most common parameter (75.6% of activations), followed by tachypnea (50.4%) and fever (48.5%), while hypothermia was present in only 13% of cases. Leukocytosis was present in 61.1% of activations, while leukopenia was observed in 11.3% of cases. Overall data are available in Table 2.

Among organ dysfunction parameters, hypotension was the most frequently observed (53%), followed by hypoxemia (50.1%), oliguria (28.9%), and altered mental status (22%). Laboratory indicators of organ dysfunction included elevated creatinine (26%), coagulopathy (19.9%), hyperlactatemia (19.8%), and hyperbilirubinemia (6.8%). On average, protocol activations were generated based on a median of 5 detection criteria (IQR: 5–7) with a median of 2 organ dysfunctions (IQR: 1–2). All dysfunction data are available in Table 2.

Based on sepsis classifications, 89% of patients presented with severe sepsis (according to Sepsis-2 criteria with at least one organ dysfunction), while 11% presented with septic shock at protocol activation, across the whole cohort. The mean number of organ dysfunctions at presentation was 2.2 in the early years of the study and 1.7 in more recent years (*p* < 0.05).

### 3.3. Hospital Location and Timeframe Activation

Regarding the location of protocol activation, 54% (n = 7828) occurred in the ED, 34.2% (n = 4970) in ICU, and 11.8% (n = 1748) in wards. Analysis of activation timing revealed that 46.5% (n = 6763) of sepsis protocols were activated during regular hospital hours (8:00–15:00), 26.3% (n = 3819) during evening hours (15:00–21:00), and 27.2% (n = 3964) during night-time hours (21:00–8:00). Table 3 shows the difference between grouped services.

### 3.4. Mortality Analysis

**Figure 1:**
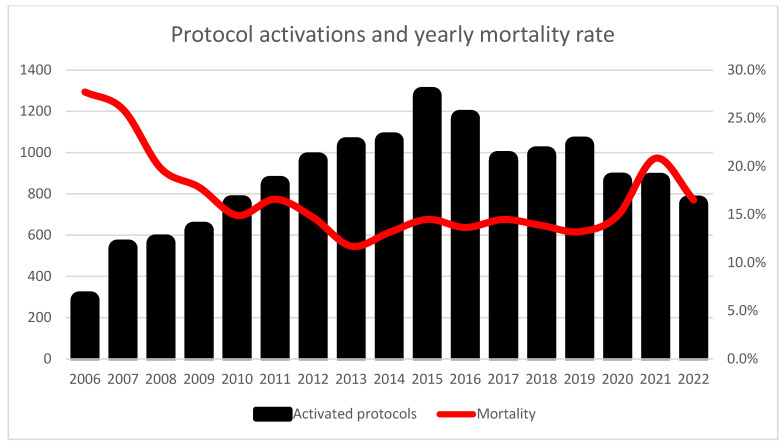
Temporal trends in sepsis protocol activations and in-hospital mortality rates from 2006 to 2022. Black bars represent the annual number of protocol activations (left *y*-axis), and the red line shows corresponding mortality rates (right *y*-axis). Notable increase in mortality during the COVID-19 pandemic period (2020–2022).

The overall in-hospital mortality for the entire cohort was 15.7%. Mortality rates varied significantly based on the location of protocol activation, with patients in the ICU having the highest mortality (24.84%), followed by wards (13.5%), and ED (10.36%) (*p* < 0.0001 between both ED and wards vs. ICU, ns. In ED vs. Ward). The highest yearly mortality was 26.5% in the first year, decreasing to 13.6% in later years (*p* < 0.01). During the COVID-19 pandemic period (2020–2022), mortality increased to 17.5%. The yearly protocol activations and mortality are shown in Figure 1.

Univariate analysis identified several clinical parameters significantly associated with increased mortality. Among SIRS criteria, hypothermia (OR 1.98; 95% CI 1.67–2.35) and leukopenia (OR 2.11; 95% CI 1.78–2.50) were strongly associated with higher mortality, while fever (OR 0.68; 95% CI 0.61–0.76) was associated with improved survival. Among organ dysfunction parameters, hyperlactatemia (OR 2.47; 95% CI 2.09–2.92), altered consciousness (OR 2.31; 95% CI 1.89–2.82), and hypotension (OR 2.13; 95% CI 1.79–2.54) were the strongest predictors of mortality. All results are synthetized in Table 4.

**Figure 2:**
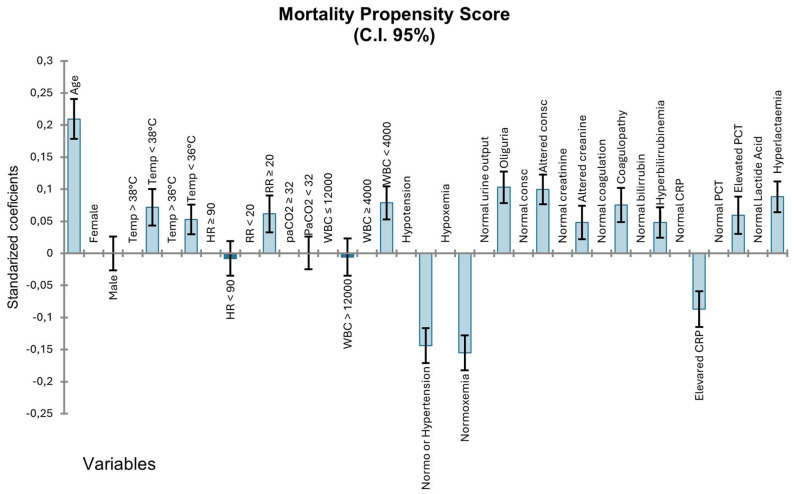
Mortality propensity score analysis for sepsis protocol activation variables. Standardized coefficients with 95% confidence intervals showing the association between activation criteria and in-hospital mortality. Positive values indicate increased mortality risk, negative values indicate a protective effect. Variables include demographic characteristics, SIRS criteria, and organ dysfunction parameters.

In multivariate analysis, adjusting for age, sex, and sepsis severity, the independent predictors of mortality were hyperlactatemia (adjusted OR 2.21; 95% CI 1.84–2.65), altered consciousness (adjusted OR 2.09; 95% CI 1.72–2.54), hypotension (adjusted OR 1.87; 95% CI 1.56–2.24), leukopenia (adjusted OR 1.79; 95% CI 1.43–2.24), hypoxemia (adjusted OR 1.52; 95% CI 1.28–1.81), and oliguria (adjusted OR 1.48; 95% CI 1.22–1.79). Patients with septic shock had significantly higher mortality compared to those with severe sepsis without shock (32.4% vs. 14.2%, *p* < 0.001). Figure 2 shows the propensity score for age, sex and activation criteria considered in the multivariate analysis.

When including in the multivariate model the number of activation criteria, ICU admission, the number of active dysfunctions, and the activation year the significant parameters for hospital mortality are age (OR 1.018, CI 1.024–1.032), length of stay (OR 1.007, CI 1.005–1.009), and ICU admission (OR 2.02, CI 1.803–2.262).

### 3.5. Resource Utilization

**Figure 3:**
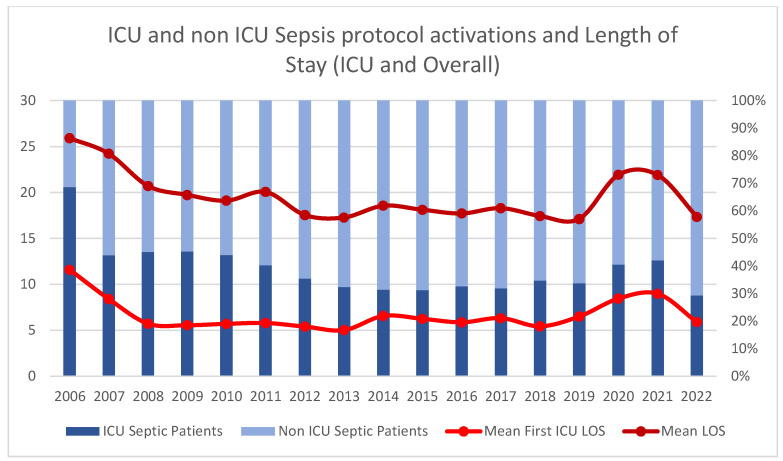
Temporal trends in ICU admission rates and length of stay for sepsis protocol activations from 2006 to 2022. Stacked bars show the proportion of ICU (dark blue) versus non-ICU (light blue) patients (left *y*-axis). Red lines represent mean ICU length of stay and overall hospital length of stay in days (right *y*-axis).

The overall ICU admission rate for patients with sepsis protocol activation was 39.7% across the entire study period, with annual variations ranging from 58% to 24%, decreasing across the years (*p* < 0.0001). The median hospital length of stay for the entire cohort was 19.0 days (IQR: 17.1–25.9), showing a general trend of reduction over the years studied (*p* < 0.0001). The median length of ICU stay for patients requiring critical care was 6.4 days (IQR: 5.0–11.6) for the entire cohort (<0.0001). Both hospital and ICU lengths of stay showed variations during the COVID-19 pandemic period (2020–2022), with significant increases observed particularly in 2020 and 2021 (*p* < 0.01). Yearly ICU admissions percentage and mean LOS are presented in Figure 3.

### 3.6. Antibiotic Administration Timing

Antibiotic administration timing data were available from 2014 onwards following the implementation of the computerized protocol system. Antibiotic initiation within 1 h of protocol activation was sustained at approximately 40% of patients from 2014 to 2020, increasing to over 60% in 2021 and 2022. Antibiotic administration within 2 h rose steadily every year from 60% in 2014 to 90% during the last two years of the study period (2021–2022).

## 4. Discussion

This comprehensive 16-year analysis demonstrates that hospital-wide sepsis protocols achieve sustained mortality reduction (from 26.5% to 13.6%) with improved resource utilization efficiency. Among 10,919 patients with 14,546 protocol activations, independent mortality predictors included hyperlactatemia (aOR 2.21), altered consciousness (aOR 2.09), and hypotension (aOR 1.87), while ICU admission rates decreased from 58% to 24% over the study period. The large sample size and extended observation period offer a robust foundation for understanding the patterns of sepsis protocol activation and the factors associated with mortality in this high-risk population.

Our experience aligns with international evidence demonstrating the effectiveness of structured sepsis protocols in reducing mortality and improving care quality across diverse healthcare systems. The New York State mandated public reporting program, encompassing 91,357 patients across 183 hospitals over 27 months, achieved a risk-adjusted mortality reduction from 28.8% to 24.4% (4.4 percentage point decrease), with improved bundle compliance from 53.4% to 64.7% for 3-h bundles and 23.9% to 30.8% for 6-h bundles [17]. Similarly, regional implementations have shown comparable benefits: the Aragón Sepsis Code, implemented across six emergency departments with 444 patients over a 3-year period, demonstrated a mortality reduction from 31.1% to 20.7% (10.4 percentage point decrease), while the Hospital La Princesa protocol, involving 1121 patients over 4 years, reported mortality declining from 24% to 15% (9 percentage point decrease) [18,19,20]. A comprehensive meta-analysis by Damiani et al., encompassing 50 observational studies with 434,447 patients, confirmed that performance improvement programs are consistently associated with reduced sepsis mortality (OR 0.66, 95% CI 0.61–0.72) and enhanced bundle compliance [14]. Our 16-year analysis represents one of the most extensive single-center sepsis protocol evaluations reported to date, with a 12.9 percentage point mortality reduction (from 37% before sepsis protocol to 26.5% in the first year, up to 13.6% in the lowest point) and sustained implementation across all hospital departments. The magnitude of mortality reduction in our cohort (49% relative reduction) exceeds that reported in most comparable studies, potentially reflecting the comprehensive nature of our multidisciplinary approach, extended implementation period, and hospital-wide scope of protocol activation, suggesting that sustained, institution-wide sepsis protocols may achieve superior outcomes compared to time-limited or department-specific interventions.

Our findings regarding the distribution of SIRS criteria at activation reveal important clinical patterns. Tachycardia was the most prevalent parameter (75.6%), followed by tachypnea (50.4%) and fever (48.5%), while hypothermia was relatively uncommon (13%). This aligns with previous studies that have identified tachycardia as one of the earliest and most sensitive indicators of sepsis, though its specificity is limited by multiple alternative etiologies [21]. The relatively low prevalence of fever highlights the importance of recognizing sepsis even in the absence of this traditionally emphasized sign, particularly in elderly populations where fever may be blunted or absent despite serious infection [22]. This observation supports the ongoing need for multifaceted screening approaches that do not rely exclusively on temperature abnormalities.

The analysis of organ dysfunction parameters offers equally valuable insights. Hypotension (53%) and hypoxemia (50.1%) were the most common manifestations, followed by oliguria (28.9%) and altered mental status (22%). These findings are consistent with the pathophysiological cascade of sepsis, where circulatory and respiratory compromise often precede dysfunction in other organ systems [23]. The relatively lower prevalence of laboratory indicators such as hyperlactatemia (19.8%) and hyperbilirubinemia (6.8%) may reflect either the timing of protocol activation before these abnormalities develop or problems in obtaining these values at the time of initial assessment, particularly in non-ICU settings.

The distribution of protocol activations across hospital locations—54% in ED, 34.2% in ICU, and 11.8% in inpatient wards—reflects the critical role of EDs as the primary point of identification for sepsis, particularly for community-acquired cases. The relatively high proportion of ICU activations suggests that sepsis recognition may be delayed in some patients until they require critical care, highlighting an area for potential improvement in early detection strategies [24]. The temporal distribution of activations, with 46.5% occurring during regular hours and 53.5% during evening and night hours, indicates that sepsis presents round-the-clock and underscores the importance of maintaining detection capabilities outside of regular working hours [25].

The multivariate analysis of mortality predictors provides a robust assessment of the factors associated with poor outcomes in sepsis. Hyperlactatemia emerged as the strongest independent predictor (adjusted OR 2.21), followed by altered consciousness (adjusted OR 2.09), hypotension (adjusted OR 1.87), and leukopenia (adjusted OR 1.79). These findings align with the established understanding that lactate elevation, neurological dysfunction, and hemodynamic compromise represent severe physiological derangements with prognostic significance [24,26]. Notably, the protective association of fever (OR 0.68) with survival supports the concept that robust inflammatory responses may confer a survival advantage [27], while hypothermia and leukopenia may represent impaired host defenses and thus correlate with poorer outcomes [28].

The observed variation in mortality rates based on location of protocol activation—24.84% in ICU, 13.5% in wards, and 10.36% in ED reflects several potential factors. Higher ICU mortality likely represents a combination of greater disease severity in this population and potential delays in recognition for patients who deteriorate after admission [29,30]. The relatively lower mortality in ED activations may reflect earlier intervention for community-acquired sepsis, the younger age and lower severity and comorbidity burden of this population, or potential differences in pathogen virulence between community and hospital-acquired infections [3].

Resource utilization patterns revealed by our analysis highlight the substantial healthcare burden associated with sepsis. The overall ICU admission rate of 39.7%, with annual variations ranging from 24% to 58%, reflects the high acuity of these patients and the significant critical care resources required for their management. The median hospital length of stay of 19.0 days and ICU stay of 6.4 days further underscore the prolonged hospitalizations often required for sepsis recovery [31,32]. A reduction in hospital length of stay has also been described in the New York implementation analysis from Levy [17]. The observed increases in both metrics during the COVID-19 pandemic period (2020–2022) are consistent with reports from other institutions and highlight the impact of this unprecedented healthcare crisis on sepsis care delivery [33].

The time-series analysis demonstrating an upward trend in sepsis protocol activations, paralleled by a decline in overall mortality rates, suggests improved recognition and management of sepsis over time. The increase in protocol activations may reflect enhanced awareness, expanded screening efforts, or changing patient demographics with increased susceptibility to infection [3] The concurrent decline in the mean number of organ dysfunctions at activation from 2.2 to 1.7 may represent earlier recognition before progression to multi-organ failure, though this interpretation requires cautious consideration of other potential explanations, including changes in documentation practices or case-mix over time [20,34].

Despite achieving significant mortality reduction, our data suggest rates have plateaued in recent years, indicating the need for next-generation interventions. The implementation of emerging technologies, particularly predictive artificial intelligence systems, represents the next logical step in sepsis care evolution. Our institution is currently developing and validating AI-driven predictive models that we anticipate will provide the breakthrough needed to overcome current mortality limitations. These systems, combined with personalized sepsis phenotyping and enhanced antimicrobial stewardship programs, may enable earlier detection and more precise interventions than traditional protocol-based approaches.

The impact of the COVID-19 pandemic is evident in our data, with increased mortality (17.5%) and resource utilization during 2020–2022. This observation aligns with global experiences during this period, where healthcare systems faced unprecedented challenges, including resource constraints, staff burnout, modified care protocols, and a surge in patients with a novel pathogen that often presented with sepsis-like syndromes [35,36,37]. A hospital-wide increase in sepsis patients’ relative mortality of 20–25% is lower than the reported increase in other similar centers [38,39], but the resilience observed in sepsis detection and management systems during such extraordinary circumstances warrants further investigation as healthcare systems prepare for future pandemics or public health emergencies.

Several limitations must be acknowledged when interpreting our findings. As a single-center study, the generalizability to other settings with different resources, patient populations, or organizational structures may be limited. The retrospective nature of the analysis introduces potential for selection bias and confounding variables, although the large sample size and extended time period mitigate some of these concerns. Changes in sepsis definitions, clinical practice patterns, and documentation approaches over the 16-year study period may have influenced the characteristics of patients identified through the protocol, though our consistent requirement for organ dysfunction helps maintain cohort homogeneity [40]. The absence of standardized comorbidity data in our database represents a limitation that may have impacted our mortality analysis. While comorbidity is a known predictor of mortality in all hospitalized patients, rather than a sepsis-specific factor, our odds ratio calculations may have reduced accuracy due to this missing information. However, it is important to note that comorbidity was considered during prospective protocol activation by clinicians, which helped limit false positives in sepsis identification, even though this information was not systematically captured in our database for analytical purposes. Additionally, we were unable to assess the appropriateness of antimicrobial therapy, which may have influenced clinical outcomes.

The consistent use of Sepsis-2 definitions throughout the study period, while methodologically necessary for temporal comparability, may limit direct comparison with contemporary studies using Sepsis-3 criteria. However, this approach was intentionally maintained to preserve the early detection focus of our protocol and avoid introducing temporal bias in our longitudinal analysis. Future studies comparing outcomes between Sepsis-2 and Sepsis-3-based protocols in similar hospital-wide implementations would provide valuable comparative data.

### Recommendations

Based on our 16-year experience with hospital-wide sepsis protocol implementation, we recommend several actionable strategies to reduce sepsis mortality.

**What must be done:**

- Implement comprehensive hospital-wide sepsis protocols with consistent activation criteria;
- Establish a 24/7 protocol activation capability across all hospital departments;
- Ensure multidisciplinary team involvement, including critical care, infectious diseases, and pharmacy specialists.

**What should be done:**

- Achieve antibiotic administration within 1 h of protocol activation in >50% of cases;
- Implement systematic staff education programs with periodic reinforcement;
- Establish continuous quality monitoring with feedback mechanisms.

**What may be done:**

- Consider integration of advanced biomarkers (PCT, PSP, MDW) for enhanced early detection;
- Explore the implementation of machine learning algorithms for automated sepsis screening;
- Develop post-sepsis syndrome management protocols.

## 5. Conclusions

The implementation of a comprehensive hospital-wide sepsis protocol demonstrates sustained reduction in sepsis mortality over a 16-year period, decreasing from 26.5% to 13.6%. The standardized evaluation criteria and systematic patient registry facilitate early risk stratification and enable timelier, targeted therapeutic interventions for high-risk sepsis patients. These findings support the implementation of comprehensive sepsis protocols as an effective strategy for improving sepsis outcomes and optimizing resource allocation in acute care settings.

## Figures and Tables

**Table 1:** Operational definitions used for sepsis protocol activation criteria throughout the study period. Abbreviations: SIRS—Systemic Inflammatory Response Syndrome; PaCO_2_—Partial pressure of carbon dioxide; PaO_2_/FiO_2_—Ratio of arterial oxygen partial pressure to fractional inspired oxygen.

Concept	Operational Definition
SIRS Criteria	Temperature > 38 °C or <36 °C heart rate > 90 beats/minute respiratory rate > 20 breaths/minute or PaCO_2_ < 32 mmHg white blood cell count > 12,000/mm3 or <4000/mm3 or >10% immature bands
Organ dysfunction criteria	Systolic blood pressure < 90 mmHg or mean arterial pressure < 70 mmHg or decrease > 40 mmHg from baseline PaO_2_/FiO_2_ < 300 urine output < 0.5 mL/kg/h for at least 2 h creatinine increase > 0.5 mg/dL from baseline international normalized ratio > 1.5 or activated partial thromboplastin time > 60 s platelet count < 100,000/mm^3^ total bilirubin > 4 mg/dL lactate > 3 mmol/L altered mental status
Sepsis	Presence of at least two SIRS criteria plus at least one organ dysfunction criterion
Septic shock	Severe sepsis with hypotension requiring vasopressor therapy despite adequate fluid resuscitation

**Table 2:** Clinical characteristics and activation criteria of patients with sepsis protocol activation stratified by severity (n = 14,546 total activations). Data are presented as percentages for categorical variables and means for continuous variables. Statistical comparisons between sepsis and septic shock groups using appropriate tests. SIRS—Systemic Inflammatory Response Syndrome; WBC—White Blood Cell count.

Item	Overall	Sepsis	Septic Shock	*p*-Value
Demographic characteristics				
Frequency	100%	89%	11%	-
Male index	60.97%	61.05%	60.23%	0.561
Age	65.75 years	65.42 years	68.99 years	<0.0001
SIRS				
Temperature > 38 °C	48.55%	50.05%	33.8%	<0.0001
Temperature < 36 °C	7.86%	7.07%	15.56%	<0.0001
Heart Ratio > 90	75.57%	75.04%	80.71%	<0.0001
Respiratory rate > 20	50.37%	49.42%	59.79%	<0.0001
pCO2 < 32	15.04%	13.7%	28.15%	<0.0001
WBC > 12.000	61.09%	61.77%	54.43%	<0.0001
WBC < 4.000	8.84%	8.15%	15.71%	<0.0001
C-Reactive Protein 2x NUL	72.6%	72.58%	72.89%	0.804
Procalcitonin 2x NUL	35.83%	34.15%	52.34%	<0.0001
Disfunctions				
Hypotension	40.52%	34.47%	100%	<0.0001
Hypoxemia	36.47%	36.36%	37.52%	0.396
Oliguria	19.0%	16.13%	48.4%	<0.0001
Altered mental status	15.32%	14.02%	28.15%	<0.0001
Elevated creatinine	25.45%	23.31%	46.54%	<0.0001
Coagulopathy	20.53%	21.59%	31.79%	<0.0001
Hyperbilirubinemia	4.83%	4.42%	8.86%	<0.0001
Hyperlacticaemia	17.02%	8.57%	100%	<0.0001
Activation criteria				
Mean activation criteria	5.57	5.3	8.15	<0.0001
Disfunctions	1.81	1.59	4.01	<0.0001

**Table 3:** Comparison of clinical characteristics and outcomes by hospital location of sepsis protocol activation (Ward n = 1748; ICU n = 4970; ER n = 7828). Data are presented as percentages and means with statistical comparisons between location pairs. ICU—Intensive Care Unit; ER—Emergency Room; LOS—Length of Stay.

Item	Ward	ICU	ER	*p*-Value
Demographic characteristics				
Male index	59.4%	63%	59.8%	ICU-ER = 0.001 ICU-Ward < 0.001 ER-Ward = 0.732
Age (years)	65.92	64.43	67.19	ICU-ER < 0.0001 ICU-Ward < 0.0001 ER-Ward < 0.0001
SIRS				
Temperature > 38 °C	50.3%	48.9%	46.7%	ICU-ER = 0.02 ICU-Ward = 0.168 ER-Ward = 0.001
Temperature < 36 °C	5.5%	11.5%	5.5%	ICU-ER < 0.0001 ICU-Ward < 0.0001 ER-Ward = 0.91
Heart Ratio > 90	72.1%	78.4%	74.9%	ICU-ER < 0.0001 ICU-Ward < 0.0001 ER-Ward = 0.003
Respiratory rate > 20	44.9%	56.2%	47.9%	ICU-ER < 0.0001 ICU-Ward < 0.0001 ER-Ward = 0.005
pCO2 < 32	15.4%	8.7%	22.2%	ICU-ER < 0.0001 ICU-Ward < 0.0001 ER-Ward < 0.0001
WBC > 12.000	63.1%	61%	59.6%	ICU-ER = 0.136 ICU-Ward = 0.034 ER-Ward = 0.001
WBC < 4.000	9.7%	10.3%	6.5%	ICU-ER < 0.0001 ICU-Ward = 0.313 ER-Ward < 0.0001
C-Reactive Protein	75.3%	76.9%	65.4%	ICU-ER < 0.0001 ICU-Ward = 0.071 ER-Ward < 0.0001
Procalcitonin	31.2%	47.1%	26.1%	ICU-ER < 0.0001 ICU-Ward < 0.0001 ER-Ward < 0.0001
Disfunctions				
Hypotension	31.9%	50.1%	36%	ICU-ER < 0.0001 ICU-Ward < 0.0001 ER-Ward < 0.0001
Hypoxemia	32.3%	41.2%	34.2%	ICU-ER < 0.0001 ICU-Ward < 0.0001 ER-Ward = 0.061
Oliguria	13.4%	29.9%	10.6%	ICU-ER < 0.0001 ICU-Ward < 0.0001 ER-Ward < 0.0001
Altered mental status	14.1%	16.3%	15.1%	ICU-ER = 0.08 ICU-Ward = 0.003 ER-Ward = 0.211
Elevated creatinine	24.3%	30.2%	20.7%	ICU-ER < 0.0001 ICU-Ward < 0.0001 ER-Ward < 0.0001
Coagulopathy	18.6%	34.3%	11.8%	ICU-ER < 0.0001 ICU-Ward < 0.0001 ER-Ward < 0.0001
Hyperbilirubinemia	4%	6.9%	3.1%	ICU-ER < 0.0001 ICU-Ward < 0.0001 ER-Ward = 0.027
Hyperlacticaemia	13%	17.8%	19.3%	ICU-ER = 0.043 ICU-Ward < 0.0001 ER-Ward < 0.0001
Activation criteria				
Mean activation criteria	5.192	6.257	5.055	ICU-ER < 0.0001 ICU-Ward < 0.0001 ER-Ward < 0.0001
Disfunctions	1.516	2.267	1.508	ICU-ER < 0.0001 ICU-Ward < 0.0001 ER-Ward = 0.498
Outcomes				
Shock	6.1%	12.9%	7.4%	ICU-ER < 0.0001 ICU-Ward < 0.0001 ER-Ward = 0.014
LOS	15.3447	28.331	11.060	ICU-ER < 0.0001 ICU-Ward < 0.0001 ER-Ward < 0.0001
Mortality	11%	23%	10.9%	ICU-ER < 0.0001 ICU-Ward < 0.0001 ER-Ward = 0.845

**Table 4:** Univariate analysis of mortality predictors in sepsis protocol activations. Results presented as Odds Ratios with 95% Confidence Intervals. Bold values indicate statistically significant associations (*p* < 0.05).

Activation Criteria	Odds Ratio (CI 95%)
Temperature > 38 °C	**0.595 (0.543–0.652)**
Temperature < 36 °C	**2.267 (1.976–2.602)**
Heart Ratio > 90	1.082 (9.742–1.202)
Respiratory Ratio > 20	**1.769 (1.614–1.939)**
PaCO2 < 32	**1.335 (1.187–1.501)**
WBC > 12.000	**0.839 (0.766–0.919)**
WBC < 4.000	**1.781 (1.553–2.042)**
C-Reactive Protein	**0.691 (0.628–0.760)**
Procalcitonin	**1.199 (1.094–1.314)**
Hypotension	**2.140 (1.955–2.343)**
Hypoxemia	**1.784 (1.630–1.952)**
Oliguria	**2.609 (2.363–2.881)**
Elevated creatinine	**1.729 (1.571–1.902)**
Coagulopathy	**1.713 (1.431–2.051)**
Hyperlacticaemia	**2.935 (2.61–2.665)**

## Data Availability

The data that support the findings of this study are available upon reasonable request to the corresponding author.

## Abbreviations

The following abbreviations are used in this manuscript:

aOR	Adjusted odds ratio
CEIC	Clinical Research Ethics Committee
CI	Confidence interval
COVID-19	Coronavirus disease 2019
ED	Emergency department
ER	Emergency room
ICU	Intensive care unit
IdISBa	Health Research Institute of the Balearic Islands
IQR	Interquartile range
LOS	Length of stay
MSU	Multidisciplinary sepsis unit
OR	Odds ratio
PaCO2	Partial pressure of carbon dioxide
PaO2/FiO2	Ratio of arterial oxygen partial pressure to fractional inspired oxygen
PIMIS	Computerized Multidisciplinary and Integral Sepsis Protocol
SD	Standard deviation
SIRS	Systemic inflammatory response syndrome
WBC	White blood cell count
